# Whole Genome Sequencing and Phylogenetic Analysis of Rabies Viruses from Bats in Connecticut, USA, 2018–2019

**DOI:** 10.3390/v13122500

**Published:** 2021-12-13

**Authors:** Ji-Yeon Hyeon, Guillermo R. Risatti, Zeinab H. Helal, Holly McGinnis, Maureen Sims, Amelia Hunt, David H. Chung, Junwon Kim, Julia Desiato, Dong-Hun Lee

**Affiliations:** 1 Connecticut Veterinary Medical Diagnostic Laboratory, Department of Pathobiology and Veterinary Science, College of Agriculture, Health and Natural Resources, University of Connecticut, Storrs, CT 06269, USA; jiyeon.hyeon@uconn.edu (J.-Y.H.); guillermo.risatti@uconn.edu (G.R.R.); zeinab.helal@uconn.edu (Z.H.H.); holly.mcginnis@uconn.edu (H.M.); maureen.sims@uconn.edu (M.S.); amelia.hunt@uconn.edu (A.H.); 2 Genomics and Molecular Epidemiology Research Laboratory, Department of Pathobiology and Veterinary Science, College of Agriculture, Health and Natural Resources, University of Connecticut, Storrs, CT 06269, USA; hyunjung.chung@uconn.edu (D.H.C.); junwon.kim@uconn.edu (J.K.); julia.desiato@uconn.edu (J.D.)

**Keywords:** rabies, bat, virus, genome sequencing, epidemiology, phylogenetic analysis

## Abstract

We performed whole genome sequencing and genetic characterization of rabies viruses (RABV) detected in bats submitted to the Connecticut Veterinary Medical Diagnostic Laboratory (CVMDL) during 2018–2019. Among 88 bats submitted to CVMDL, six brain samples (6.8%, 95% confidence interval: 1.6% to 12.1%) tested positive by direct fluorescent antibody test. RABVs were detected in big brown bats (*Eptesicus fuscus, n* = 4), a hoary bat (*Lasiurus cinereus, n* = 1), and an unidentified bat species (*n* = 1). Complete coding sequences of four out of six detected RABVs were obtained. In phylogenetic analysis, the RABVs (18-62, 18-4347, and 19-2274) from big brown bats belong to the bats EF-E1 clade, clustering with RABVs detected from the same bat species in Pennsylvania and New Jersey. The bat RABV (19-2898) detected from the migratory hoary bat belongs to the bats LC clade, clustering with the eleven viruses detected from the same species in Arizona, Washington, Idaho, and Tennessee. The approach used in this study generated novel data regarding genetic relationships of RABV variants, including their reservoirs, and their spatial origin and it would be useful as reference data for future investigations on RABV in North America. Continued surveillance and genome sequencing of bat RABV would be needed to monitor virus evolution and transmission, and to assess the emergence of genetic mutations that may be relevant for public health.

## 1. Introduction

Rabies is the oldest known zoonotic disease caused by any of the 17 recognized viruses of the genus Lyssavirus; family Rhabdoviridae [1]. It is now considered a re-emerging disease in different countries worldwide and responsible for an estimated 59,000 human deaths annually [2]. Rabies lyssaviruses are found in the Americas, Europe, Asia, Africa, and Australia, and at least 30 reservoir species have been identified, consisting primarily of carnivorous mammals and bats [3,4,5]. Of the *Lyssavirus* species, rabies virus (RABV) is the only one naturally present in the Americas, and only on this continent do bats act as RABV hosts [6,7].

In the United States, since national canine rabies control efforts began in earnest in the early 1940s, the primary reservoir species responsible for maintaining RABV have been bats, raccoons, striped skunks, gray foxes, arctic foxes, and mongooses [4]. The majority of domestically acquired human rabies cases in the United States are caused by bats. Among over 1300 different species of bats in the world, there are 47 species of bats in the United States, and nine species of bats can be found in the state of Connecticut; big brown bat, little brown bat, northern long-eared bat, eastern small-footed bat, Indiana bat, tri-colored bat, silver-haired bat, eastern red bat, and hoary bat [8,9]. All species except the big brown bat are on Connecticut’s List of Endangered, Threatened and Special Concern Species [9]. According to the rabies surveillance in the United States during 2018 [4], a total of 27,483 bats were tested in the country, of which 1635 (5.9%) were confirmed positive for rabies. Bats were the most frequently reported rabid animals representing 33.0% (*n* = 1635 of 4951) of all animal rabies cases. In addition, among humans with rabies acquired in the United States, bat RABV variants accounted for most deaths during 1960–2018; 62 (70%) of 89 acquired human rabies cases [10,11]. Therefore, rabies in bats remains a major public health concern.

Although bats are frequently reported rabid animals, there is limited complete genome sequence data and whole genome sequence (WGS) phylogenetic analysis of RABVs from bats. In a previous study, Kuzmin et al. reported WGS of 15 RABV from bats, including nine RABV from big brown bats during 2001–2010 in the Flagstaff area of Arizona and six historical big brown bat RABV isolates obtained during 1975–1999 [12]. As of 26 July 2021, only 35 complete genome sequences of bat RABVs in United States between 1975 and 2010 have been reported in NCBI GenBank database. Furthermore, there is no report in the database regarding WGS of bat RABV detected in the New England region.

The virus typing methods including antigenic typing and phylogenetic typing have been used for investigating bat RABVs, such as the identification of the source of rabies infection in humans and livestock in the absence of records of either a bat bite or bat activity in a particular area [13]. Phylogenetic classification of RABV variants has been based on partial gene sequences indicative of specific genes such as nucleoprotein (N) and glycoprotein (G) genes [6,14,15]. However, WGS techniques allow for a more accurate phylogenetic classification on the evolution, spread, and genome wide heterogeneity of viruses [16,17]. For example, using WGS, Brunt et al. [16] revealed the most probable origins of three raccoon RABV variants from three animals that had unexpectedly broken into a rabies-free zone. In this study, we detected RABVs from bats submitted for rabies testing to the Connecticut Veterinary Medical Diagnostic Laboratory (CVMDL) between the years 2018–2019 as a passive surveillance. We report the first complete coding sequences of the bat RABVs in the New England region and their phylogenetic relationship with other bat RABVs recovered from across the United States.

## 2. Materials and Methods

### 2.1. Bat Samples and Rabies Diagnostic Tests

During 2018–2019, a total of 88 bats were submitted for laboratory testing for rabies to the CVMDL, Department of Pathobiology and Veterinary Science, University of Connecticut. We used brain tissue samples to diagnose rabies using direct fluorescent antibody (DFA) test [18]. By signing the CVMDL’s rabies testing request form, submitters agreed that CVMDL reserves the right to utilize these clinical specimens as anonymized material for teaching and research purposes.

### 2.2. RNA Extraction and SISPA

The presence of the viral RNA was confirmed by using the quantitative reverse transcription real-time PCR (RT-qPCR) assay [19]. A sample was not available for further analysis because there was no brain tissue left after DFA. Total RNA was extracted from five rabies-positive remaining brain tissue samples after DFA using the TRIzol reagent (ThermoFisher Scientific, Waltham, MA, USA) according to manufacturer’s instructions. Sequence-Independent, Single-Primer-Amplification (SISPA) was performed to amplify viral RNA as described in the previous study [20]. Briefly, first-strand cDNA was synthesized in a 20 µL reaction mixture with 5 µL of total RNA from each sample, 100 pmol of primer K-8N (GACCATCTAGCGACCTCCACNNNNNNNN), SuperScript IV Reverse Transcriptase (ThermoFisher Scientific), and dNTPs (10 µM) following the manufacturer’s instructions. To convert the first-strand cDNA into double-stranded cDNA, 20 µL of the first-strand cDNA was heated to 95 °C for 3 min and then cooled to 4 °C in the presence of 10 pmol of primer K-8N, and 10 µM dNTPs in 1× Klenow reaction buffer (New England Biolabs, Ipswich, MA, USA). Afterwards, 1 uL of Klenow fragment were added and incubated at 37 °C for 60 min (final volume, 25 µL). After conversion into dsDNA by Klenow polymerase (New England Biolabs), the products were purified using Agencourt AMPure XP beads (Beckman Coulter, Brea, CA, USA). The purified dsDNA of the Klenow reaction was subsequently used as a template for PCR amplification. PCR amplification was conducted with 5 µL of the double-stranded cDNA template in a final reaction volume of 50 µL, which contained 1× Phusion HF buffer, 200 μM deoxynucleoside triphosphate (dNTP), 10 µM primer K (GACCATCTAGCGACCTCCAC), and 0.5 U Phusion DNA polymerase (New England Biolabs). The PCR cycling was performed as follows: 98 °C for 30 s, followed by 35 cycles of 98 °C for10 s, 55 °C for 30 s, and 72 °C for 1 min, with a final extension at 72 °C for 10 min. PCR products were purified using Agencourt AMPure XP beads (Beckman Coulter). For quantification of the amplified dsDNA, the Qubit dsDNA HS assay (ThermoFisher Scientific) was performed according to the manufacturer’s instruction.

### 2.3. Whole Genome Sequencing

The Swift 2S Turbo DNA Library Kit (Swift Sciences, Ann Arbor, MI, USA) was used according to the manufacturer’s instructions to generate multiplexed paired-end sequencing libraries of four samples. The dsDNA was fragmented and tagged with adapters by Nextera transposase. Sequencing libraries were purified using Agencourt AMPure XP beads (Beckman Coulter) and analyzed on a High Sensitivity DNA Chip on the Bioanalyzer (Agilent Technologies, Santa Clara, CA, USA). The libraries were adjusted to 1 nM concentration and equal volumes of 5 µL of each library were pooled. The pool was denatured with NaOH (0.2 N final concentration) and further diluted to 100 pM. Five percent of PhiX control library (Illumina, Foster City, CA, USA) was added to the pool. The library pool was loaded in the flow cell of the 151 cycle ISeq100 i1 Reagent Kit (Illumina). The barcoded multiplexed library sequencing (2 × 151 bp) was performed on an Illumina iSeq100 platform (Illumina).

### 2.4. Assembly of Sequencing Reads

De Novo and reference guided genome assemblies against reference genome sequences (GenBank accession number: JQ685920 and JQ685947) were performed using the Geneious Prime 10 Software (https://www.geneious.com/, accessed on 1 September 2021) and Galaxy instance [21]. We uploaded all fastq files generated in this study to Galaxy and utilized batch mode to recognize and run pairs together. Residual adapters and bases with low quality scores were removed from fastq files using Trimmomatic version 0.38.0 with conservative parameters, which included removing bases from each read with a quality score < Q 30 and required a minimum read length of 100 bases each. Trimmed reads were assembled and the consensus genome sequences were called using the Geneious Prime 10 with default parameter settings.

### 2.5. Bat Species Identification

DNA barcoding based on the mitochondrial gene cytochrome c oxidase subunit 1 (cox1) has been previously applied to studies of bat rabies for identification of host species [22]. We confirmed the bat species of each sample by DNA barcoding based on the cox1 gene. We obtained a consensus genome of cox1 gene for each sample by reference guided genome assembly of NGS reads to reference mitochondrial genome sequences (GenBank accession number: NC_029346, GU722958, and MF143474) using Geneious Prime 10 Software. Species-level identification was performed using the BARCODE OF LIFE DATA SYSTEM v4 (https://www.boldsystems.org/, accessed on 25 October 2021).

### 2.6. Phylogenetic Analysis

A total of 192 N gene sequences and 35 WGS of RABVs identified in bats in the United States were downloaded from NCBI GenBank database. The software MAFFT multiple alignment Version: 1.4.0 in Geneious was used for sequence alignment of whole genome and N gene sequences. Maximum likelihood (ML) phylogenies were constructed using RAxML-HPC v.8 with 1000 bootstrap replicates on XSEDE with default parameter settings [23]. RABV-GLUE (http://rabv-glue.cvr.gla.ac.uk/#/rabvFastaAnalysis accessed on 1 September 2021) was used for the automated genotyping analysis of the major/minor clade.

## 3. Results and Discussion

**Figure 1:**
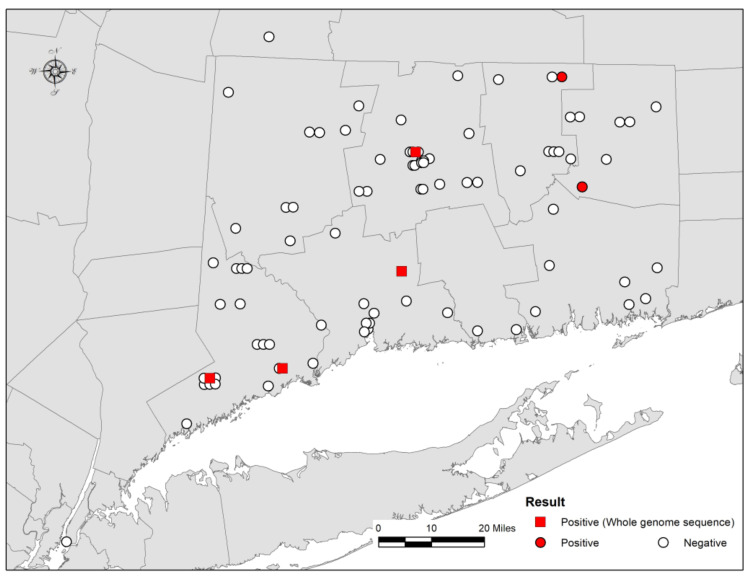
Geographic distribution of bats submitted to Connecticut Veterinary Medical Diagnostic Laboratory (CVMDL) for laboratory testing for rabies in Connecticut, the United States, during 2018–2019.

A total of 88 bats were submitted to CVMDL for rabies testing with 86 bats originating from the state of Connecticut, one bat from the state of New York, and one bat from the state of Massachusetts. In Connecticut, bats were collected in all eight counties including Fairfield (*n* = 21), Hartford (*n* = 22), Litchfield (*n* = 9), Middlesex (*n* = 2), New Haven (*n* = 10), New London (*n* = 7), Tolland (*n* = 8) and Windham (*n* = 7) (Figure 1 and Table S1). RABV was detected in six brain samples (6.8% of the total) by the DFA test (Figure 1 and Table 1). RABV-positive samples originated from Fairfield County (*n* = 2), Hartford County (*n* = 1), New Haven County (*n* = 1), Tolland County (*n* = 1), and Windham County (*n* = 1) (Figure 1 and Table S1). According to Rabies surveillance data in the United States, the proportion of rabid bats (6.8%, 95% confidence interval: 1.6% to 12.1%) observed at CVMDL during 2018–2019 is similar to the proportions observed by others during the period 2010–2013 (6.0%) [10], 2013–2017 (6.3%) [4], and 2018 (5.9%) [4] suggesting a steady proportion of rabid bats in the country.

All five samples were RABV positive in RT-qPCR with threshold cycle (Ct) values from 21.05 to 36.0. Complete coding sequences of RABV were obtained from four samples with Ct values of <29.39 (Table 2). By using the SISPA and WGS, we were able to obtain not only the viral genome sequences but also cox1 gene sequences for species identification. The host species of the five positive samples were identified as big brown bat (18-62, 18-3792, 18-4347, and 19-2274) and hoary bat (19-2898) by DNA barcoding (Table 1). Big brown bat is the most frequently tested bat species in the United States [4,10]. We assume that it may be due to their habitat preferences which there is more likelihood of contact with humans and domestic animals than other bat species [24]. It may result in a greater proportion of healthy animals being found and submitted for testing. Nonetheless, the RABV positive rates in big brown bats has been lower relative to other bat species; 3.6% (2269 out of 62,997 tested) during the period 2010–2015 [10] and 4.2% (377 out of 9064 tested) during the year 2018 [4]. Interestingly, the RABV positive rates in migratory hoary bats has been significantly higher than those in big brown bats, with 142 RABV positive bats out of 342 tested (46.0%) in the United States for the period encompassing 2010 to 2015 [10]. Similar trends were observed for the 2018 survey of bat RABV when 49.4% of the hoary bats (39 out of 79 tested) tested positive for RABV [4]. In the United States, the highest RABV positive rates were observed among Mexican free-tailed bats (*Tadarida brasiliensis*) with a 52.3% (1050 out of 2006 tested) for the period 2010–2015 [10], and among canyon bats (*Parastrellus hesperus*) with a 60.3% (41 out of 68 tested) in 2018 [4]. According to the U.S. national rabies surveillance system [4], 35 rabid wild animal cases were reported in the state of Connecticut during 2018 including 15 rabid raccoon cases and 14 rabid bat cases. These data suggest that bats together with raccoons are most likely prominent rabies reservoir species in the state of Connecticut.

**Figure 2:**
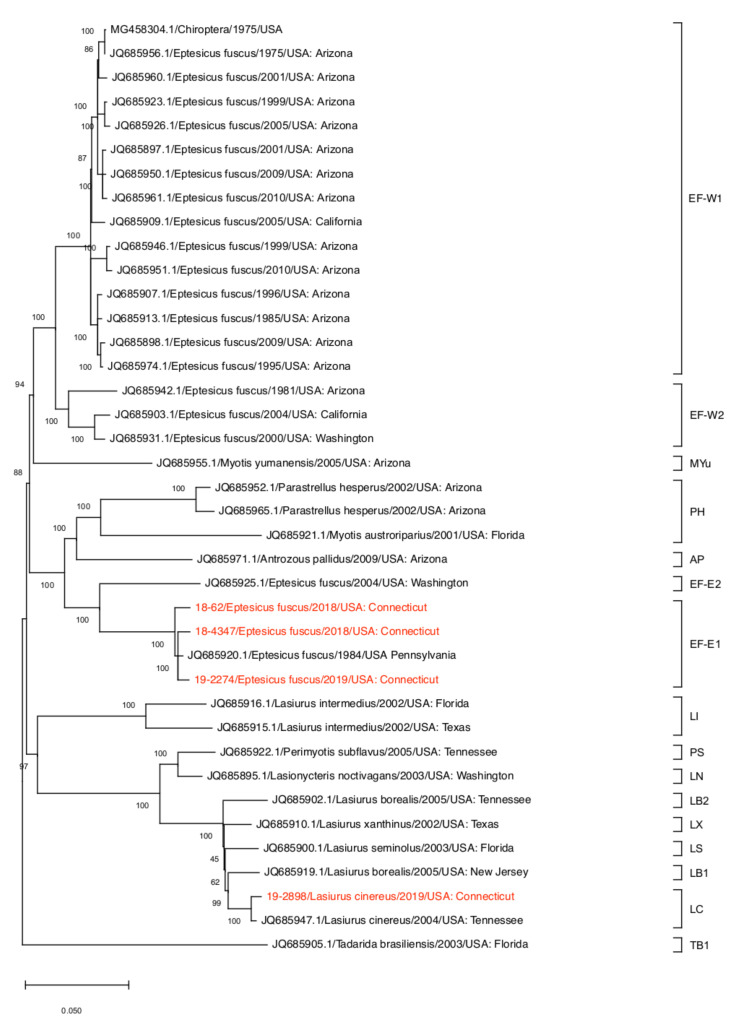
Maximum-likelihood analysis of 39 complete coding sequences of bat RABVs identified in the United States including four RABVs of this study. The phylogeny was rooted at midpoint. The scale bars show the number of substitutions per site. The numerical values represent 1000 bootstrap replicate values expressed as a percentage.

The ML phylogenetic trees based on the complete coding sequences and the N gene sequences of RABVs detected in the United States available in the GenBank are shown in Figure 2 and Figure S1, respectively, with the genetic clades assigned by RABV-GLUE. The phylogenetic trees show similar topology for the four RABV sequenced in this study (18-62, 18-4347, 19-2274, and 19-2898). The 18-62, 18-4347, and 19-2274 RABVs detected in big brown bats were assigned to the bats EF-E1 clade, clustering together with the RABVs detected from the same species of bats in Pennsylvania in 1984 and 4 RABVs from New Jersey (Figure 2 and Figures S1 and S2). In contrast, the 19-2898 RABV detected in a hoary bat was assigned to the bats LC clade, clustering with 11 RABVs detected mostly from the same species of bat in Arizona, Washington, Idaho, and Tennessee, and two RABVs detected in other bat species including, a silver-haired bat (*Lasionycteris noctivagans*) in Idaho and western yellow bat (*Lasiurus xanthinus*) in California (Figure 2 and Figures S1 and S2).

While RABV detected in terrestrial animals tend to associate with their geographical origins [16,25,26], our phylogenetic analysis suggests that most distinct clades of RABV affect different species of bats in the United States. In the phylogenetic analysis of N gene that include all sequences of bat RABVs identified in the United States available in the GenBank as of 19 October 2021, the bats AP, bats EF-E1, bats EF-W2, bat LI, and bats MYu clades consist of one bat species in each clade (Figure S1 and Table S2). Most RABVs from big brown bats in our analysis were assigned to multiple clades, including bats EF-E1, bats EF-E2, bats EF-W1, and bats EF-W2 (Table S2). Consistent with this finding, it has been suggested that RABV in nonmigratory bat species, such as big brown bats, tend to have higher genetic variation and geographic clustering of lineages compared to migratory bats (Figure S2) [26]. On the other hand, the 19-2898 RABV from hoary bat detected in this study showed >99% sequence identity with the RABV detected in hoary bats in Arizona, Washington, Idaho, and Tennessee (Figures S1 and S2). RABV in migratory bat species such as hoary bats follow their host species throughout its migration range [26]. Since hoary bats can make long-distance and multi-directional movements during their migratory season [27], we assume that the widespread detection of highly similar RABV in hoary bats is the result of viral transmission during long-distance migratory flights of infected bats.

In this study, we report passive surveillance results for bat RABV in the state of Connecticut during 2018–2019 and announce the first complete genome sequences of bat RABVs in the state. The complete genome sequences and their phylogenetic relationships with other bat RABVs established in this study would be useful as reference data for future investigations on RABV in North America. Continued surveillance and genome sequencing of bat RABV would be needed to monitor virus evolution and transmission, and to assess the emergence of genetic mutations that may be relevant for public health.

## Figures and Tables

**Table 1:** Number of bats submitted to Connecticut Veterinary Medical Diagnostic Laboratory (CVMDL) for laboratory testing for rabies in Connecticut, the United States, during 2018–2019.

Year	Bat Species	No. of Bats Tested for Rabies	No. of RABV Positive Samples (Sample ID)
2018	*Eptesicus fuscus*	8	3 (18-62, 18-3792, 18-4347)
	*Lasionycteris noctivagans*	1	0
	Unidentified	44	1 *
2019	*Eptesicus fuscus*	13	1 (19-2274)
	*Lasiurus borealis*	1	0
	*Lasiurus cinereus*	2	1 (19-2898)
	*Myotis lucifugus*	2	0
	Unidentified	19	0
Total		88	6

* The sample was not available for genome sequencing.

**Table 2:** The Ct values of the RT-qPCR and the summary of the whole genome sequencing results.

Sample	Ct Value	Total NGS Reads	Q/C Passed	Assembled Reads (%)	Complete CDS (Mean Depth of Coverage)	Identified Species by DNA Barcoding (Top Hit %)
18-62	26.29	797,418	687,856	3108 (0.45)	Yes (37.4)	Big brown bat (99.85%)
18-3792	36.0	726,524	609,074	14 (0.003)	No (0.1)	Big brown bat (100%)
18-4347	24.57	607,824	53,2362	3887 (0.73)	Yes (47)	Big brown bat (99.17%)
19-2274	21.05	722,642	638,616	2348 (0.37)	Yes (28.4)	Big brown bat (99.85%)
19-2898	29.39	661,854	578,494	2459 (0.43)	Yes (29.6)	Hoary bat (100%)

## Data Availability

The complete coding genome sequences of the RABVs in this study have been deposited in the Genbank under accession number OK340700-OK340703.

## Supplementary Materials

The following are available online at https://www.mdpi.com/article/10.3390/v13122500/s1, Table S1: List of bat samples tested for rabies submitted to Connecticut Veterinary Medical Diagnostic Laboratory (CVMDL) between 2018–2019. Table S2: Number of RABV sequences used for the phylogenetic analysis of N gene, by bat species and clades by RABV-GLUE. Figure S2: Geographic distribution of genetic clades of bat RABVs in the United States, based on N gene sequences available in the GenBank, assigned by RABV-GLUE. Figure S1: ML phylogeny of 196 bat RABV N gene sequences from the United States with four RABVs sequenced in this study. The tree was rooted at midpoint. The scale bar shows the number of substitutions per site. The numerical values represent 1000 bootstrap replicate values expressed as a percentage.
