# Supplementary material for: Whole Genome Sequencing and Phylogenetic Analysis of Rabies Viruses from Bats in Connecticut, USA, 2018–2019
Source: Viruses. 2021 Dec 13;13(12):2500. doi: 10.3390/v13122500 (PMC8704678; doi:10.3390/v13122500)
Supplement: Supplementary file 1 [file viruses-13-02500-s001.zip › Figure S1.pdf]

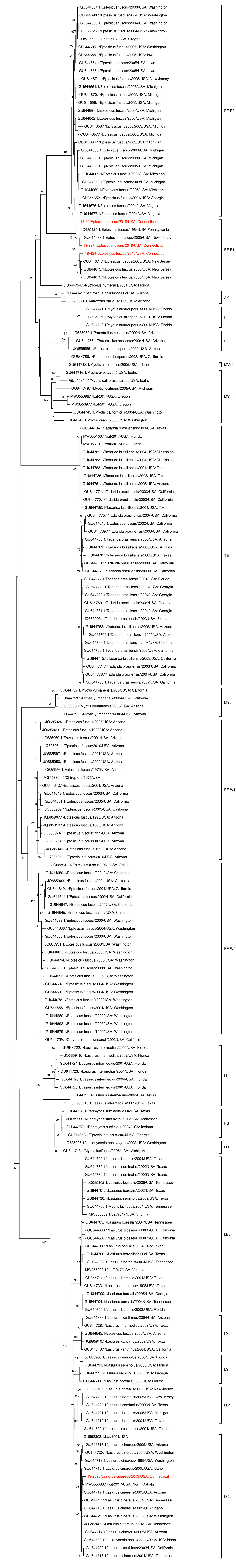

Figure S1: 196 N sequences of bat RABVs isolated in United States including four RABVs of this study (the phylogeny was rooted at midpoint, the scale bars show the number of substitutions per site, the numerical
