# Supplementary material for: Whole Genome Sequencing and Phylogenetic Analysis of Rabies Viruses from Bats in Connecticut, USA, 2018–2019
Source: Viruses. 2021 Dec 13;13(12):2500. doi: 10.3390/v13122500 (PMC8704678; doi:10.3390/v13122500)
Supplement: Supplementary file 1 [file viruses-13-02500-s001.zip › Figure S2.pdf]

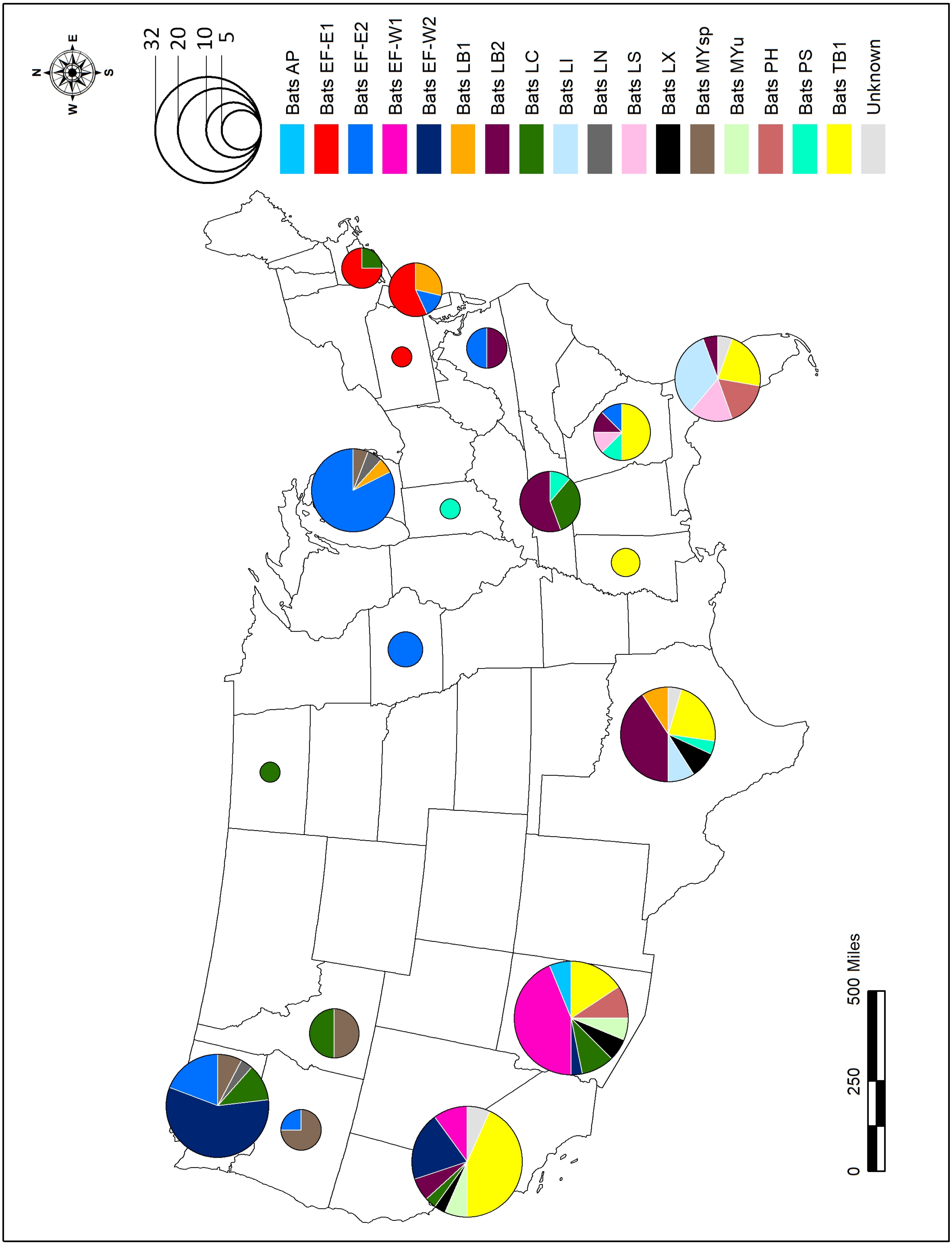

Figure S2: Geographic distribution of genetic clades of bat RABVs in the United States, based on N gene sequences available in the GenBank, assigned by RABV-GLUE.
